# Altered plasticity of the parasympathetic innervation in the recovering rat submandibular gland following extensive atrophy

**DOI:** 10.1113/expphysiol.2008.045112

**Published:** 2008-11-21

**Authors:** G H Carpenter, N Khosravani, J Ekström, S M Osailan, K P Paterson, G B Proctor

**Affiliations:** 1 Salivary Research Unit, Floor 17, Tower Wing, Kings College London Dental InstituteLondon SE1 9RT, UK; 2 Department of Pharmacology, Gothenburg University, Sahlgrenska AcademyMedicinaregatan 15 D, POB 431, Gothenburg, S-41390, Sweden

## Abstract

Adult rat submandibular glands have a rich autonomic innervation, with parasympathetic and sympathetic nerves working in synergy rather than antagonistically. Ligation of the secretory duct rapidly causes atrophy and the loss of most acini, which are the main target cell for parasympathetic nerves. Following deligation, there is a recovery of gland structure and function, as assessed by autonomimetic stimulation. This study examines whether the parasympathetic nerves reattach to new target cells to form functional neuro-effector junctions. Under recovery anaesthesia, the submandibular duct of adult male rats was ligated via an intra-oral approach to avoid damaging the chorda-lingual nerve. Four weeks later, rats were either killed or anaesthetized and the ligation clip removed. Following a further 8 weeks, both submandibular ducts were cannulated under terminal anaesthesia. Salivary flows were then stimulated electrically (chorda-lingual nerve at 2, 5 and 10 Hz) and subsequently by methacholine (whole-body infusion at two doses). Glands were excised, weighed and divided for further *in vitro* studies or fixed for histological examination. Ligation of ducts caused 75% loss of gland weight, with the loss of most acinar cells. Of the remaining acini, only 50% were innervated despite unchanged choline acetyltransferase activity, suggesting few parasympathetic nerves had died. Following deligation, submandibular glands recovered half their weight and had normal morphology. Salivary flows from both glands (per unit of gland tissue) were similar when evoked by methacholine but greater from the deligated glands when evoked by nerve stimulation. This suggests that parasympathetic nerves had reattached to new target cells in the recovered glands at a greater ratio than normal, confirming reinnervation of the regenerating gland.

The ligation of the rat submandibular gland duct has been studied extensively because it causes severe atrophy of the gland with time (Ohlin & Perec, 1967; Tamarin, 1979), which can be reversed if the ligature is removed from the duct (Tamarin, 1971*a*,*b*;). This remarkable ability of the gland to regenerate allows the investigation of innervation mechanisms normally only present during the embryonic and neonatal development of salivary glands. Following ligation, as atrophy develops, acinar cell volume reduces, axon length is reduced and nerves appear to lose contact with salivary cells (Garrett *et al.* 1979). The total level of choline acetyltransferase, a good marker for parasympathetic nerves, is also reduced, suggesting fewer cholinergic nerves (Banns *et al.* 1979). The present study tests whether deligation of the ducts and the recovery of acinar cells allows the parasympathetic nerves to reform the neuro-effector junctions that are essential for normal function of the salivary reflex in conscious animals (including humans).

Deligation of the submandibular duct allows the salivary gland to recover size and function. By using an intra-oral route to perform the ligation, damage of the chorda-lingual nerve supplying the submandibular gland (Osailan *et al.* 2006), which might compromise any study on the remaining innervation, is avoided. To assess neuro-effector junctions we use salivary secretion *in vivo*, and for *in vitro* studies a novel combination of electrical field stimulation (EFS) of nerves with the recording of acinar intracellular calcium fluxes.

## Methods

All animal procedures were conducted with approval of the local Animal Ethics and Welfare Committee and under a Home Office project licence. Animals were killed by an overdose of pentobarbitone.

### *In vivo* experiments

#### Ligation and deligation

Four adult male Wistar rats (Harlan Laboratories, Loughborough, UK) weighing 275–325 g were placed under recovery anaesthesia (ketamine, 75 mg kg^−1^ and xylazine, 15 mg kg^−1^, i.p.) and a metal clip placed on the right submandibular duct through a small incision in the floor of the mouth which was then sutured. Four weeks later, rats were placed under terminal anaesthesia (pentobarbitone 48 mg kg^−1^_I.P._, followed by chloralose, 80 mg kg^−1^i.v.) for the procedures detailed below. In a second group of five rats, ligation was carried out as described but after 4 weeks clips were removed from the submandibular duct under recovery anaesthesia (ketamine, 75 mg kg^−1^ and xylazine, 15 mg kg^−1^, i.p.). Animals were left for a further 8 weeks and under terminal anaesthesia (anaesthetic overdose) the following procedures were carried out. Rats were killed by anaesthetic overdose (200 mg kg^−1^ pentobarbitone).

#### Parasympathetic nerve stimulation

The chorda-lingual nerve for each gland was cut deeply and reflected onto the submandibular duct. Following cannulation of the duct with plastic tubing (0.28 mm), both nerve and duct were placed in a bipolar electrode and stimulated at 2, 5 and 10 Hz (2 ms, 5 V) for 5 min each with 1 min intervals. Saliva samples were collected on ice into preweighed tubes to calculate salivary flow rates.

#### Autonomimetic infusions

An intravenous cannula (0.28 mm) was inserted into the right femoral vein during induction of anaesthesia and this was connected to a calibrated syringe pump, as previously described (Proctor *et al.* 2003). For the experiments described in this paper, methacholine was continually infused at a dosage of 4 or 12 μg min^−1^ (kg body weight)^−1^. Saliva samples were collected from both left and right submandibular ducts simultaneously.

### General histology and nerve assessments

Fixed submandibular tissue was embedded in paraffin wax and 10 μm sections were stained with Haematoxylin and Eosin for general morphology. Alcian Blue/Periodic Acid Schiff's (AB/PAS) stain was used to show secretory granules in acinar cells and *p*-dimethylaminobenzoaldehyde (DMAB) to specifically show kallikrein-containing granules in granular tubules as previously described (Garrett *et al.* 1996). A cholinesterase-specific substrate was used to stain parasympathetic nerves as previously described (Bogart, 1970; Garrett & Anderson, 1991).

Choline acetyltransferase activity was assessed as described previously (Khosravani *et al.* 2006) in glandular homogenates. Activities are expressed as concentrations and multiplied by gland weights for total activity.

### *In vitro* experiments

Following ligation as described above, glands were removed from anaesthetized rats (*n*= 11) and digested by collagenase (5 mg in 20 ml buffer) as previously described (Carpenter *et al.* 2004). Cells from the collagenase-digested glands were loaded with the calcium-sensitive dye fluo 4 AM (4 μm; Invitrogen, Paisley, UK). With a constant flow of buffer, cells were electrically stimulated by a pair of platinum wires as previously described by Gallacher (1983), with slight modifications. Cells were stimulated three times with 50 V, 2 ms at 50 Hz in 15 s trains and then stimulated by superfusing with increasing bursts of methacholine (from 10^−8^ to 10^−6^m) and finally ionomycin (13 μm) for the maximal signal to calibrate levels of dye within each cell. Fluorescence levels were assessed using a confocal microscope (TCS SP2, Leica, Hemel Hempstead, UK) and expressed relative to the maximal signal during ionomycin perfusion. Only cells responding by above baseline changes in intracellular calcium to methacholine, which directly stimulates the acinar cell, were included in the results. Acinar cells that also responded to electrical stimulation, which stimulates the nerve to release acetylcholine, were scored as being connected to parasympathetic nerves.

### Statistics

Results are expressed as means ±s.e.m. Multiple statistical comparisons were made using one-way ANOVA, whilst two-sample comparisons were made using Student's paired or unpaired *t* tests, and *P* values < 0.05 were considered significant.

## Results

### Glandular structure

**Figure 1:**
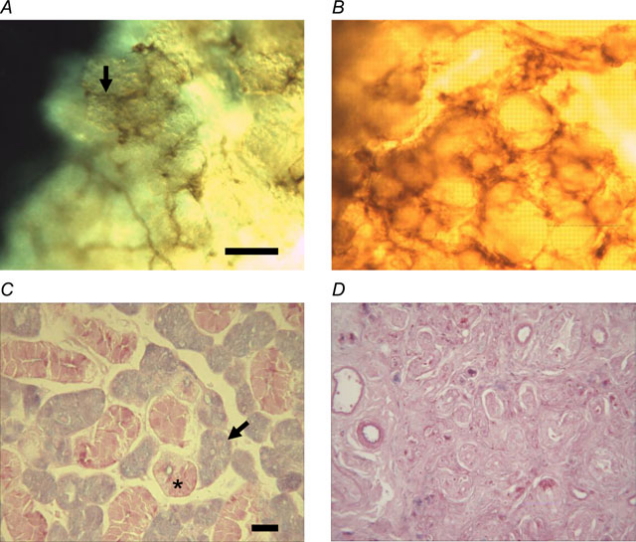
Control and ligated submandibular glands Nerve staining of collagenase-digested cell clumps from control (*A*) and 4-week-ligated glands (*B*). Cholinesterase nerve staining indicates that parasympathetic nerves (arrow in *A*) are still attached following collagenase digestion. Alcian Blue/Periodic Acid Schiff's staining of tissue sections reveals blue acinar cells (arrows) and pink granular ducts (*) in normal glands (*C*) and an almost complete loss of secretory granules in 4-week-ligated glands (*D*). Scale bar represents 30 μm.

**Figure 2:**
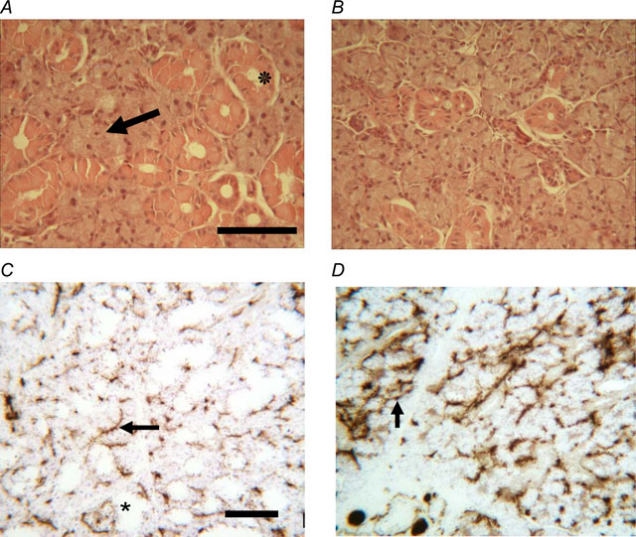
Control and ligated–deligated submandibular glands Haematoxylin and Eosin staining indicates that acini (arrow) and granular ducts (asterisk) in the control gland (*A*) and in the ligated–deligated glands (*B*) have recovered most of their volume, although granular ducts may be less numerous. Cholinesterase staining of control (*C*) and ligated–deligated glands (*D*) indicates that parasympathetic nerves (arrow) are denser and more heavily stained in the recovered gland. Note that negative staining (*) in (*C*) relates to granular ducts. Scale bar represents 100 μm.

Ligation of the submandibular duct for 4 weeks caused a significant reduction (approximately 75%) in gland size (Table 1) with a dramatic change to glandular structure. Figure 1 reveals a great reduction in numbers of acini and differentiated ducts (such as glandular, intercalated and striated) although ductal structures are still apparent, and AB/PAS, which stains the glycosylated secretory granules (Fig. 1), indicates that almost all cells have lost their secretory granules. Removal of the ductal obstruction after 4 weeks (in a different group of rats) resulted in a significant recovery in gland size (75% increase) compared with the 4-week-ligated glands. The recovery in weight reflected a recovery of both acinar and differentiated ductal structures with a reduction in undifferentiated ducts (Fig. 2*B*). Although AB/PAS staining showed that acinar cells had recovered much of their secretory material, granular ducts were not as replete with secretory granules as control glands; this was confirmed by kallikrein-specific DMAB staining (results not shown).

**Table 1:** Gland weights and choline acetyltransferase (CAT) activity

	Unoperated control	Ligated (4 weeks)	Ligated (4 weeks) and deligated (8 weeks)
Gland weight (g)	0.31 ± 0.02	0.08 ± 0.02^a^	0.14 ± 0.01*
CAT (nm g^−1^)	778 ± 28	2332 ± 401	1448 ± 98*
Total CAT (nm)	243 ± 14	176 ± 39	201 ± 8

a Mean ±s.e.m. (*n*= 5) except ligated group (mean ±s.d., *n*= 4).

* *P* < 0.05 compared with contralateral control gland.

### Salivary flow rates

**Figure 3:**
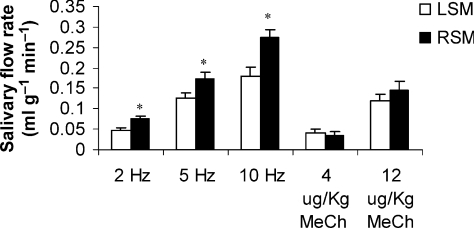
Stimulated salivary glands Graded electrical stimulation of the parasympathetic nerves supplying unoperated (LSM) and the ligated–deligated glands (RSM) reveal significant (*P < 0.05) increases in salivary flow rates when expressed per gram of secretory tissue. Whole-body methacholine stimulation (at two doses) in the same rats (*n*= 5) indicates equal salivary secretion.

Graded electrical stimulation of parasympathetic nerves supplying control and recovered submandibular glands elicited greater salivary flows (when expressed per unit of secretory tissue) from the recovered gland compared with the contralateral unoperated control gland. The increased salivary flow was on average 50% greater for each of the three stimulation frequencies (Fig. 3). In contrast, whole-body methacholine stimulation resulted in similar flow rates for the control and recovered submandibular glands at both a low and high dose, chosen to be broadly comparable to the nerve-evoked flow rates. Collection of saliva from ligated glands (collection tube posterior to ligation point) following either nerve or methacholine stimulation resulted in no recoverable saliva, even after extensive periods of stimulation.

### Parasympathetic nerve staining

Staining for cholinesterase, a useful marker for parasympathetic nerves, showed a rich innervation of control submandibular glands, particularly around acini, whilst granular ducts had a faint appearance and were sparsely innervated (Fig. 2*C*). In ligated glands, a dense staining was observed (Fig. 1*B*), which was not specifically localized to any one structure. In ligated–deligated glands, a similar dense innervation was noted, although some acinar and ductal structures were now apparent (Fig. 1*D*). The dense innervation observed using the cholinesterase staining on tissue sections was supported by measurments of the activity of choline acetyltransferase (CAT), another marker of parasympathetic nerves, in glandular homogenates (see Table 1). Ligated glands had nearly three times the concentration of control glands, and recovered glands still had double the concentration of control glands. This presumably reflects the size of the glands, since total amounts of choline acetyltransferase were similar between the three glandular conditions.

### *In vitro* responses

Finally, electrical field stimulation was used to test whether parasympathetic nerves were still attached to acini isolated from the ligated glands by observing elevated intracellular calcium, the main intracellular messenger for fluid secretion. To avoid dying/dysfunctional cells, only cells that could also be stimulated subsequently by high doses of methacholine were included in the results shown in Table 2. Control glands yielded a high proportion of cells which could also be stimulated by EFS (at the frequency used). Cells from a 1-week-ligated submandibular gland indicated a significant reduction of cells responding to EFS, although this was no longer a significant decrease after 2 weeks of ligation. The use of cells from glands with longer periods of ligation was more difficult because the tissue became progressively fibrotic and more resistant to collagenase digestion.

**Table 2:** *In vitro* assessment of nerve to acinar cell connection as number of cells showing changes in intracellular calcium in response to EFS and methacholine stimulation

	Unoperated control	1 week ligated	2 weeks ligated	4 weeks ligated
Percentage of acinar cells connected to nerves^a^	75.4 ± 7.3	43.1 ± 10.3*	64.6 ± 11.3	50.1 ± 6.9
Number of cells^b^	92 (4)	107 (3)	122 (3)	23 (1)

a Percentage of cells responsive to electrical field stimulation which also responded to methacholine stimulation.

b Number of rats.

* *P* < 0.05 compared with control cells.

## Discussion

The autonomic innervation of salivary glands has to adapt to changes in the size of the parenchymal tissue. Salivary glands are stimulated by taste and chewing, and their size can reflect changes in the diet; for instance, rat parotids can halve in weight when feed is changed from a hard to a soft chow of equal calorific value (Schneyer *et al.* 1992). Parotid and submandibular glands can also increase in size after removal of salivary duct obstruction. In order to maintain a salivary flow, the autonomic nerves must adapt and reinnervate parenchymal cells as the gland changes in size. The present report demonstrates that reinnervation of recovering salivary cells can occur following ligation–deligation. The significantly greater nerve-stimulated salivary flows from the ligated–deligated glands compared with the unoperated contralateral glands suggest that a greater number of parasympathetic nerves have reattached to each secretory acinus, probably as a result of the reduced number of acini reattaching to the same number of nerves. Following longer periods of deligation, salivary glands can recover almost to their full size (Osailan *et al.* 2006); however, with the shorter periods used in this study there was not a full recovery of size and so salivary flows were expressed per gram of secretory tissue. The assumption that equal weights of glandular tissue reflect equal secretory capacity appears valid, since whole-body methacholine stimulation produced equal flows (per gram) from the two glands (ligated–deligated and contralateral control). Histologically, the recovered gland looked normal, with acinar volume appearing similar to control glands although granular ducts were not totally replete with secretory granules, which may reflect their slower rate of secretory protein resynthesis (Proctor, 1999).

In the present study, essentially no secretion could be evoked from ligated submandibular glands, and this observation has previously been described. Other studies have shown that secretory function of acinar cells is greatly decreased within 24 h of ligation (Carpenter *et al.* 2007), and all secretory granules disappear after 2–3 days (Tamarin, 1971*b*), at which time apoptosis peaks (Takahashi *et al.* 2000). By 7–14 days of ligation, few acinar cells can be seen (Tamarin, 1971*b*; Cotroneo *et al.* 2008), and by 4 weeks of ligation glands appear atrophic, having lost over half their normal weight (Tamarin, 1971*b*). Reabsorption of ions by striated ductal cell function is similarly affected early in ligation (Carpenter *et al.* 2007), and granular ductal cells also degranulate after 2–3 days (Tamarin, 1971*a*). Unlike acini, however, ductal cells start to proliferate during ligation (Takahashi *et al.* 2000), forming long undifferentiated (i.e. not discernable as striated, granular or intercalated) ductal structures, which may provide the support for the nerves in an otherwise dying glandular structure.

Our study of nerves and their function provides new information that may answer the question of how the ligated gland recovers from extensive atrophy. Despite the loss of the finer nerves that normally innervate acini there still remained extensive cholinesterase staining. To remove the possibility that nerves appeared thicker and denser as an artefact of a shrunken tissue, quantification of the nerves was achieved by measuring glandular choline acetyltransferase activity, a useful marker for parasympathetic nerves (Ekström *et al.* 1977). Greater concentrations of choline acetyltransferase were detected in ligated compared with control glands, coinciding with the increased cholinesterase staining of tissue sections even though total choline acetyltransferase activity levels revealed only a slight decrease. Longer periods of ligation do appear to cause a loss of nerves; after a 3 week ligation period, total choline acetyltransferase activity was decreased by 20% (Banns *et al.* 1979), and a 50% loss of neurones was noted with 8 weeks of ligation (Womble & Roper, 1987). At the time point used (4 weeks) in the present study, however, we conclude that most parasympathetic nerves remain intact.

Since ligation causes an immediate reduction of salivary secretion, as judged by whole-body autonomimetic stimulation (Carpenter *et al.* 2007), a different method was needed to assess whether nerves were still functionally attached to any remaining acini. Following activation of cholinergic receptors by parasympathetic nerves (Proctor, 2006), intracellular calcium fluxes in acinar cells are vital for the activation of ion channels and the creation of an osmotic gradient for salivary flow. Despite cessation of salivary flow, intracellular calcium fluxes were found to be normal in acinar cells from a ligated parotid gland (Liu *et al.* 1998). Therefore, in a new combination of techniques, we used EFS to stimulate nerves still attached to acinar clumps *in vitro* and assessed functional neuronal junctions by monitoring intracellular calcium flux in acinar cells. Firstly, we established in cells from unoperated adult submandibular glands that collagenase digestion did not separate nerves from salivary cells by staining nerves in clumps of collagenase-digested glands. Intimate nerve staining was apparent, and it was even possible to see nerve endings on some acini (Fig. 1*A*), which are difficult to see in tissue sections. Although EFS is a relatively inefficient method to stimulate nerves (compared to direct contact), it was possible to stimulate the majority of acinar cells which were also responsive to methacholine stimulation. The fact that all cells did not respond to EFS probably reflects the low efficiency of EFS and acinar cell turnover. It is unknown how many cells are normally in contact with nerves, presumably most, but at any one time 5–10% of cells may have recently divided (Ihrler *et al.* 2004) or may be in the process of differentiating into acinar cells (Denny *et al.* 1997) and therefore not responsive to stimulation. It might be hypothesized that glandular atrophy would lead to a complete detachment of parasympathetic nerves from acinar cells, but that does not appear to have occurred. Electrical field stimulation with intracellular calcium imaging suggests that over half of the remaining acini are still attached to nerves. Overall, given the combined reduction in the numbers of acini responding to EFS and methacholine and the loss of the majority of acinar cells (Tamarin, 1971*b*; Takahashi *et al.* 2000) with ligation, it must be concluded that there are few acinar cells remaining that are connected to parasympathetic nerves in the ligated gland.

The greater nerve-evoked salivary flows (per gram tissue) from the ligated–deligated gland clearly indicate a reinnervation of acinar cells in the recovering gland. From the ligated state, in which there are many nerves but few acini, the recovering (Takahashi *et al.* 2004) and newly developing acini (Cotroneo *et al.* 2008) become innervated, probably by releasing as yet undefined neurotrophic factors. By comparison with other models, Ectodysplasin (Hinck, 2004) and Wingless/Int (Wnt) pathways (Zou, 2004) may be involved, and all these molecules are expressed in developing embryonic salivary glands (Hoffman *et al.* 2002). Whatever the neurotrophic factors, interaction with the large numbers of axons present in the ligated gland produced a greater than normal innervation of recovering acini, hence a greater flow of saliva with nerve stimulation. Salivary cell innervation does not involve specialized neuro-effector junctions, such as those of muscle, and so more than one nerve can contact each target cell. Hypolemmal and epilemmal relationships ensure that nerves are close enough to basolateral membranes of acinar cells (Garrett, 1999). In normal glands, each acinus can be innervated by more than one axon; however, in the regenerated glands it would appear possible that the deligated acini became innervated with an even greater number of axons, a phenomenon shown previously (Ekström & Emmelin, 1971). Thus, the greater salivary flows might result from each acinus receiving a greater dose of acetylcholine at a given frequency of electrical stimulation than the control glands.

In conclusion, this study has shown the remarkable ability of the salivary glands to recover from extensive atrophy to become functional, (and presumably) reflexly secreting salivary glands, and that the degree of innervation of parenchymal cells was dependent on target cells and axon numbers. In the ligated gland, there was increased nerve density relative to acini; however, EFS/intracellular calcium did not indicate an increased innervation, but rather a decrease. Thus, timing of neurotrophic factors and relative densities of target and nerve cells appear to determine innervation patterns in rat salivary glands.
